# Clinical and Radiologic Characteristics, Surgical Outcomes, and Its Possible Origins of Chondroma of the Dural Convexity

**DOI:** 10.1155/2020/5961358

**Published:** 2020-12-17

**Authors:** Jing Guo, Qiuyue Fang, Jianhua Cheng, Chuzhong Li, Songbai Gui, Yazhuo Zhang, Lei Cao

**Affiliations:** ^1^Beijing Neurosurgical Institute, Capital Medical University, Beijing, China; ^2^Department of Neurosurgery, Beijing Tiantan Hospital Affiliated to Capital Medical University, Beijing, China; ^3^Beijing Institute for Brain Disorders Brain Tumor Center, Beijing, China; ^4^China National Clinical Research Center for Neurological Diseases, Beijing, China; ^5^Key Laboratory of Central Nervous System Injury Research, Beijing, China

## Abstract

Chondroma of the dural convexity (CDC) is a benign and extremely rare type of intracranial chondroma. In this study, we reported five CDCs in a single center and reviewed the available literature to determine the clinical characteristics and surgical outcomes and possible origins of the disease. The clinical data of five patients (4 females) who confirmed to be CDC between 2000 and 2019 in our single center was collected together with 22 cases from literatures. The clinical characteristics and surgical outcomes were reviewed and analyzed. Among all the available CDC cases, the mean age was 31 ± 13.7 years; the mean tumor volume was 42.3 ± 40.9 cm^3^, showing a female predominance (63% vs. 37%). The tumors showed calcification in 88.2% cases (15/17) on CT scans and hypointense on T1WI (15/19, 78.9%), mixed intense on T2WI (10/18, 55.6%), and inhomogeneous enhancement without dural tail sign after administration of gadolinium (20/21, 95.2%). Almost all the tumors were misdiagnosed as meningiomas preoperatively. In addition, almost all image available CDC lesions (24/25, 96%) located across the cranial sutures indicating that the tumor originated from ectopic chondrocytes from adjacent skull sutures. No tumors recurred after total resection in follow-up. CDCs are characterized with female predominance and may originate from ectopic chondrocytes from adjacent skull sutures. The lesion with inhomogeneous contrast enhancement without dural tail sign and avascular in cerebral angiography are key points to be differentiated from meningioma. The most effective treatment is total resection.

## 1. Introduction

Chondromas are benign, slow-growing tumors that originate in cartilage [1]. These tumor cells are mainly composed of cartilaginous cells and chondrocytes, which can produce a cartilaginous matrix [2]. They can be found in several parts of the body, mainly in short tubular bones, especially the metacarpal and phalangeal bones [1, 3], accounting for 20% to 50% of benign bone tumors [4, 5]. Intracranial chondromas are extremely rare, with an estimated incidence of 0.2% to 0.3%, and were first reported by Hirschfeld in 1851 [6–8]. Intracranial chondromas are usually located at the base of the skull, such as the sphenoethmoidal, sphenopetrosal, spheno-occipital, or petro-occipital regions. However, approximately 15-20% of intracranial chondromas arise supratentorially from the dura, usually in a parafalcine frontoparietal location. They may grow from cartilage rests within the dura mater of the convexity or falx [2, 4, 8–10]. Only a few cases of chondromas of the dural convexity (CDCs) have been reported thus far.

In current study, by reviewing the medical records of 5 cases of CDCs from our single center and by fully reviewing the literature, we can attain a more comprehensive understanding of the common clinical characteristics, treatment strategies, and neurosurgical experience of CDCs. In addition, based on the fact that most lesions are close to the adjacent cranial suture, we can speculate on the possible origin of the chondroma.

## 2. Materials and Methods

**Figure 1:**
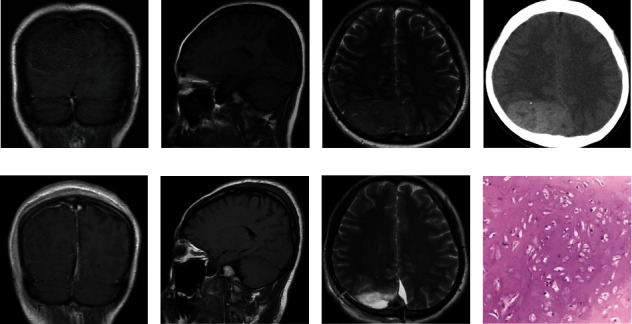
Radiographic images and hematoxylin-eosin staining of Case 1. (a–d) Pre- and (e–g) postoperative radiographic images indicated a mass located in the right parietal area with no cystic degeneration. (a) Coronal T1-weighted images (after gadolinium enhancement) demonstrate mild multiple dot and linear enhancement. (b) Sagittal T1-weighted images demonstrate a slightly hypointense signal. (c) Axial T2 demonstrates a slightly hyperintense signal with no significant edema or mass effect in adjacent structures. (d) CT scan shows that the lesion was hyperdense with calcification. (e–g) Postoperative images show that the tumor was completely removed. (h) Hematoxylin-eosin staining of Case 1.

Data from 5 CDC patients (4 female) who were surgically treated and pathologically confirmed at Beijing Tiantan Hospital, Capital Medical University, from 2000 January to 2019 July were reviewed. We extracted information about the patient's gender, age at diagnosis, clinical neurologic dysfunction, surgical procedure, and pathology from the medical records. In addition, we evaluated the tumor location, volume, boundary, and enhancement by radiologic images (Figure 1 and Fig. S1-4). The outcomes of postoperative treatment, functional status, and tumor recurrence were collected. The Karnofsky performance scale (KPS) was used to assess the patient's pre- and postoperative neurologic status. This retrospective study was approved by the institutional review board, and all subjects provided informed consent.

A tailored surgical approach based on tumor location was used to remove the tumors. The extent of tumor resection was evaluated based on pre- and post-MR and/or CT images. The extent of tumor resection was categorized as gross total resection (GTR) and non-GTR (presence of tumor residuals). Postoperative specimens were sent for pathologic examination (Figure 1 and Fig. S1-4).

“Chondroma” and “dural convexity” were searched as key words in PubMed. Any location and histopathologically confirmed CDC case report was included, limited to English publications without data restrictions. In addition, all references provided in the identified publications and included relevant citations were further reviewed. This study included cases with sufficient clinical data reported in English for analysis.

## 3. Results

### 3.1. Patient Features

In our series, the mean age at diagnosis was 42.2 ± 13.5 years (range 23-57 years, median 41 years) (Table 1). Besides combining with 22 cases in the published literature, we found the mean age was 31 ± 13.7 years (range 14-57 years, median 31 years) and female predominance (63% versus 37%) (Table 2). Most of the CDC patients present symptoms like lime numbness, dizziness, and headache induced by the mass effect (location, increased intracranial pressure) but can be asymptomatic (Table 3).

### 3.2. Radiological Manifestations

According to the preoperative imaging, most lesions of CDCs (14/27) are located in the frontal area, and their mean tumor volumes are 42.3 cm^3^ ± 40.9 (range 1.8-144.0 cm^3^). Excluding the 9 cases not reported, 8 cases (8/18, 44.4%) had hyperdensity, 7 cases (7/18, 38.9%) had mixed density, and 3 cases (3/18, 16.7%) had hypodensity on CT. Half of lesions had cystic degenerations. Moreover, we found most lesion (15/17, 88.2%) calcified on the CT scans. In MRI, fifteen cases (15/19, 78.9%) were hypointensive on T1WI, and 10 cases (10/18, 55.6%) presented mixed intensitive on T2WI. After administration of gadolinium, 20 lesions (20/21, 95.2%) were enhanced inhomogeneously on T1WI with no dural tail sign (Table 2). Almost all patients were preoperatively misdiagnosed with meningiomas.

### 3.3. Relationship between Tumor and Cranial Sutures

Studies by Maruyama and others have shown that suture mesenchymal stem cells play an important role in craniofacial bone development, repair, and regeneration [11]. Therefore, we hypothesized that the formation of CDCs may be related to such stem cells in the cranial suture. Based on the available imaging data of the 5 cases from our single center and 20 literature review cases (excluding two image not available cases) [6, 8, 10, 12–30], we found that almost all CDC lesions (24/25, 96%) were located across the cranial sutures. In our series, 4 CDCs were located across the coronal suture, and 1 was located across the parieto-occipital suture (Figure 1 and Fig. S1-4). In addition, we summarized the relationship between the lesions and adjacent cranial sutures in all available cases in Table 3. Excluding two cases without preoperative imaging in literatures, approximately 19 CDCs (19/25, 76%) were close to the coronal suture, and 5 CDCs (5/25, 20%) were close to the other sutures (Table 2). These results indicated that tumor formation may be related to the adjacent cranial sutures.

### 3.4. Surgical Outcomes and Prognosis

All patients underwent craniotomy to remove the tumors, and surgical approaches are described in Table 3. According to detailed data from our single center, all tumors had distinct boundaries with distinguishable surgical boundaries, and all 5 patients achieved GTR. Pathological and histological features were similar in the available 27 cases and showed the features of chondroma. Hematoxylin-eosin staining showed that the tumor was mainly composed of chondrocytes, similar to normal cells, and produced a cartilage matrix.

The neurological symptoms were improved in all patients when discharged. As for our series, the mean recent KPS was 98 ± 4.5, which is significantly higher than the preoperative KPS of 84 ± 5.5 (*p* < 0.01, *t*-test). In addition, none of the patients received any further adjuvant therapy, such as radiotherapy or chemical therapy. After mean and median follow-up durations of 96.8 and 112 months, respectively, no patients suffered from tumor recurrence, which is same as the all available patients' report.

### 3.5. Case Illustration

A right parieto-occipital space-occupying lesion was initially misdiagnosed as a convex meningioma in a 37-year-old woman (Case 1). She had intermittent headaches and dizziness for more than 7 years. Neurological examination revealed negative signs. An MRI scan showed a slight hypointensity in the T1-weighted imaging. T2-weighted imaging showed a slight hyperintensity, and intratumor vessels were observed in the enhanced lesion. The enhanced scan showed inhomogeneous enhancement, and the right lateral ventricular occipital angle was deformed (Figure 1). And the patient was diagnosed as meningiomas preoperatively.

Based on the MRI and CT images, we found that the lesion was located across the parieto-occipital suture, which was confirmed intraoperatively. During the operation, the lesions were clearly defined within the surrounding brain tissue. The tumor was separated along the arachnoid membrane between the tumor and brain tissue and completely removed. Upon histopathological examination, the tumor was diagnosed as a chondroma. Moreover, the immunohistochemistry examination of Case 1 showed positive results for vimentin protein, S-100, and Ki-67 (0-1%) and negative results for cytokeratin (CK), epithelial membrane antigen (EMA), Brachyury, and P53 in the tumor. There was no recurrence reported by the 1-year follow-up.

## 4. Discussion

Intracranial chondromas are very rare and benign neoplasms, accounting for less than 1% of all intracranial lesions. They can occur alone or as part of Ollier disease or Maffucci syndrome [31]. Isolated tumors usually occur at the base of the skull and tend towards the sphenoid ethmoid region [32], while chondromas of the dural convexity are less common [8]. In this study, we reported cases of dural-based convex chondroma. To our knowledge, fewer than 30 cases of dural chondroma have been reported previously [6, 18, 28]. These tumors occur less frequently than other skull-based chondromas, accounting for only 15-20% of all intracranial chondromas [21].

Intracranial dural-based convex chondroma is rare. In our current study, only 5 cases were reported. Combined with the 22 cases in the published literature, we have comprehensively summarized CDCs, including clinical and radiological characteristics, histopathology, differential diagnosis, treatment strategies, and possible pathogenesis (Table 3). Several studies have been reported that CDCs generally occurs in patients between 20 and 60 years of age with no gender predominance [9], and the incidence peaks around the third decade [12]. However, according to our data, CDCs are more predominant in females (63% versus 37%) (Table 2).

Generally, bone is formed by endochondral or intramembranous ossification. The base of the neurocranium forms through endochondral ossification [33], whereas the vault (calvarium) is thought to develop exclusively through intramembranous ossification [34]. Therefore, a number of authors believe that most chondromas at the base of the skull are formed from cartilage in the basilar synchondrosis [6, 7, 18, 22, 31, 32]. However, as the calvarium is formed from intramembranous ossification, it is difficult to explain the formation of dural convex chondroma in this part of the skull. However, Zhao et al. suggested that craniofacial sutures provide a unique niche for mesenchymal stem cells (MSCs) for craniofacial bone homeostasis and repair [35]. In addition, suture mesenchymal stem cells have been reported to play a crucial role in craniofacial bone development and regeneration [11]. In summary, the existing cases demonstrated that almost all chondromas are related to the adjacent cranial suture. Therefore, we hypothesized that the mesenchymal stem cells located in the cranial sutures differentiate into chondrocytes for some reason and thus abnormally grow into chondroma through the ectopic dura mater.

Various theories have been proposed for the occurrence of intracranial chondroma, and there are reasonable explanations. The speculative origin of dural convex chondroma reported in the literature can be divided into two categories. One is that ectopic chondrocytes enter the meninges during early development [36] or after traumatic brain injury [25]. The second origin is metaplasia of the connective tissue of the meninges (such as meningeal fibroblasts [32]) or metaplasia of perivascular mesenchymal cells [23], which may be related to inflammation or trauma. Nevertheless, the etiology remains unknown [6].

Although many theories about the origin of intracranial chondroma have been proposed, the pathogenesis of the disease is still controversial. It has been reported that systemic chondromatosis conditions may be related to intracranial chondromas [37]. Chondromatosis is usually referred to as Maffucci syndrome (multiple enchondromatosis associated with soft tissue angiomas) [38] or Ollier disease (multiple polysystemic enchondromatosis) [31]. IDH1 and IDH2 are genes that encode isocitrate dehydrogenase 1 and isocitrate dehydrogenase 2, respectively. Chondroma formation in patients with Maffucci syndrome and Ollier disease has been associated with somatic mosaic mutations in these two genes in studies by Pansuriya et al. [39, 40] and with heterozygosity mutations in PTHR1 [41] in studies by Couvineau et al. [42]. Yoshiki et al. [30] showed that there are no mutations in IDH1 and IDH2 in chondroma of the dural convexity. However, his study confirmed the expression of the full-length HMGA2 transcript but not the truncated transcript (exons 1-3) in CDCs, which may be associated with cell differentiation in meningeal chondromas, similar to soft tissue and skeletal chondromas in other areas [43]. However, there are no other reports of genetic etiologies for chondroma of the dural convexity.

Because the lesions grow slowly and do not invade brain tissue, CDCs usually have a long history of clinical symptoms [44, 45]. The lesions are usually very large in size when they are found [21]. The clinical manifestations mainly depend on the location of the lesion or increased intracranial pressure, such as headache, dizziness, and limb numbness [46]. Table 3 shows the clinical symptoms of the current existing cases.

The neuroimaging features of CDC lesions are quite typical. CT scans show variable densities due to differences in the degree of calcification. Due to the increase in lesion size, some cases may also show hypertrophy or erosion and damage to adjacent bones [18, 21]. In some cases, there is necrosis or cystic degeneration in the tumor center, resulting in a low density [6]. According to our cases, we found 50% lesion showed cystic degeneration. Besides, most lesions (88.2%) showed calcification on the CT scans, and 44.4% of cases had hyperdensities, and 38.9% of cases had mixed-densities. Lacerte et al. [26] proposed a radiological classification of chondroma. A typical chondroma is defined as a type I tumor, which appears to be iso-dense and homogeneous on CT scan, while the degenerative cyst in the tumor center is defined as a type II tumor, and the CT image shows a low density in the center of the lesion [26].

MRI showed no tissue edema around the tumor, which means that the lesions grew slowly and indicated the benign nature of the lesions [6]. These lesions appear as hypo-, iso-, or mixed intensities on the T1-weighted image and as hypo-, mixed, or uneven hyperintensities on the T2-weighted image. There was a slight circular or unevenness enhancement after administration of gadolinium [9, 47]. In our present study, 78.9% cases were hypointense on T1WI, and 55.6% cases showed mixed-intensities on T2WI. In addition, 95.2% cases showed inhomogeneous enhancement after intravenous contrast injection on T1WI with no dural tail sign. Angiograms show that CDCs are nonvascular tumors [9, 47]. In addition, CDCs are typically DWI hypointense with high ADC (apparent diffusion coefficient) values [12, 16].

The histological features of chondromas are lobules of hyalinized cartilage usually containing one cell per lacuna, and neoplastic chondrocytes remaining in the cavity are cytologically benign. Around the tumor, the cartilage undergoes endochondral ossification, and the center is often calcified. Lesions of myxoid degeneration can also be seen [6, 7, 18, 22, 32, 48].

Although the radiological and pathological characteristics are almost the same, the convexity dura-based chondroma can be distinguished from the skull base chondroma by the patients' clinical signs and symptoms.

CDCs are difficult to differentiate from meningiomas [45]. Almost all CDCs were preoperatively misdiagnosed as meningiomas. However, the clinical features are different, and patients with meningioma are older than those with CDCs [26]. Radiologically, meningiomas show a more pronounced uniform contrast enhancement with marked dural tail signs, while CDCs usually do not [12]. Due to the high cell density and compactness, meningiomas appear hyperintense on DWI and have reduced ADC values, while chondromas usually appear hypointense on DWI and with high ADC values [12, 49]. In addition, meningiomas are hypervascular in cerebral angiography, while chondromas are avascular [50].

Since CDCs are avascular lesions and do not invade or adhere to the surrounding tissue, the standard surgical strategy for these tumors is total resection [9, 27]. Furthermore, removal of the dural sheath or attachment is recommended [6, 15, 31, 48]. Most reports do not recommend radiotherapy for nonsurgical patients or postoperative residual tumors, as chondroma does not respond well to radiation and may undergo malignant transformation [9]. A number of authors believe that no recurrence or a good long-term prognosis should be expected [6, 20, 45, 48]. Considering the slow growth of the lesions and their benign nature, when CDCs are found by accident in elderly patients, observation and waiting can be used [14].

## 5. Conclusion

In this study, we found that chondromas of the dural convexity occur more frequently in females than in males. In addition, the lesion is close to the adjacent cranial suture, and we speculate that the occurrence of CDCs may be related to ectopic chondrocytes in the cranial suture. Due to easily misdiagnosed as meningioma, the lesion with inhomogeneous contrast enhancement without dural tail sign and avascular in cerebral angiography are key points to be differentiated. For chondroma of convex origin, it is expected that total surgical resection is more readily available and that the patient has no recurrence after surgery.

## Figures and Tables

**Table 1:** Clinical characteristics of current 5 cases of CDCs in our series.

Clinical characteristics		*n* (%)
Age (years)		
	<30	1 (20.0)
	≥30	4 (80.0)
	Median&mean ± standard deviation	41&42.2 ± 13.5
Gender		
	Male	1 (20.0)
	Female	4 (80.0)
Volume (cm^3^)		
	Mean ± standard deviation (cm^3^)	67.9 ± 54.2
	Range (cm^3^)	3.6-131.0
Quality of life		
	Preoperative KPS	84.0 ± 5.5
	Recent KPS	98.0 ± 4.5
Surgical resection		
	GTR	5 (100)
	Non-GTR	0
Follow-up (months)		
	Range	7-136
	Mean ± standard deviation	96.8 ± 51.2
	Median	112
	Recurrence	0

CDC: chondroma of the dural convexity; GTR: gross total resection; KPS: Karnofsky performance scale.

**Table 2:** Radiographic characteristics of 27 CDCs in previous and current studies.

Radiographic characteristics		*n* (%)^∗^
Age (years)		
	<30	13 (48.1)
	≥30	14 (51.9)
	Median&mean ± standard deviation	31&31.89 ± 13.66
Gender		
	Male	10 (37.0)
	Female	17 (63.0)
Involved regions		
	Frontal	14 (51.9)
	Fronto-parietal	6 (22.2)
	Fronto-temporal	3 (11.1)
	Parietal	3 (11.1)
	Parietal parasagittal	1 (3.7)
Volume (cm^3^)		
	Mean ± standard deviation (cm^3^)	42.3 ± 40.9
	Range (cm^3^)	1.8-144.0
CT density		
	NA	9
	Hyper	8 (44.4)
	Hypo	3 (16.7)
	Mixed	7 (38.9)
CT calcification		
	NA	10
	Calcification	15 (88.2)
	Noncalcification	2 (11.8)
Cystic degeneration		
	NA	3
	Yes	12 (50)
	No	12 (50)
MRI features		
T1WI		
	NA	8
	Hypo	15 (78.9)
	Mixed	2 (10.5)
	Iso	1 (5.3)
	Hyper	1 (5.3)
T2WI		
	NA	9
	Hyper	6 (33.3)
	Hypo	2 (11.1)
	Mixed	10 (55.6)
Enhancement		
	NA	6
	Inhomogeneous	20 (95.2)
	None	1 (4.8)
Adjacent cranial suture		
	NA	2
	Coronal suture	19 (76.0)
	Other sutures	5 (20.0)
	None	1 (4.0)

NA: not available; T1WI: T1-weighted imaging; T2-WI: T2-weighted imaging. ^∗^% = [available cases/(total cases − not available cases)] × 100%.

**Table 3:** Clinical information of available studies of CDCs.

Study	Age/gender	Preoperative symptoms/interval of symptoms, months	Location	Size	CT features	MRI Features			Cystic degeneration	Surgical approach	IHC	Preoperative diagnosis	Adjacent cranial suture	Recurrence
						T1WI	T2WI	Enhancement						
Nakayama et al. 2001 [6]	47/F	Motor weakness at left lower extremity/24	Right frontal	About 8.0 cm long	Hypodense/calcification	Hypo	Hyper	Inhomogeneous	Yes	Bifrontal craniotomy	S-100 (+)	NA	Coronal suture	NA
Erdogan et al. 2006 [8]	14/M	Seizures/1	Left frontal	6.0 × 7.0 × 4.0	Mixed density/calcification	Hypo	Hyper	Inhomogeneous	Yes	NA	NA	Meningioma	Coronal suture	NA
Cosar et al. 2005 [10]	21/F	Headache and dizziness/NA	Left frontal	2.0 × 2.0 × 2.0	Mixed density/no calcification	Hypo	NA	NA	No	Left frontoparietal craniotomy	NA	NA	Coronal suture	NA
Kawabata et al. 2012 [12]	48/F	Episodic headaches/NA	Left frontoparietal	1.5 × 1.5 × 6.3	Hyperdense/calcification	Hypo	Mixed	Inhomogeneous	Yes	Left frontoparietal osteoplastic craniotomy	S-100 (+)	Meningioma	Coronal suture	NA
Abeloos et al. 2012 [13]	22/F	Progressive headaches and cervical pain/12	Right frontoparietal	7.5 × 5.0 × 3.0	NA	Hypo	NA	Inhomogeneous	Yes	Right frontoparietal craniotomy	S-100 (+), AE1AE3 (-), EMA (-), MIB-1/Ki67 (< 1%)	Meningioma	Sagittal suture	NA
Feierabend et al. 2018 [14]	25/M	Seizure/NA	Right frontal	4.2 × 5.4 × 3.7	Mixed density/calcification	Hypo	Mixed hypo and hyper	Inhomogeneous	No	Right frontal osteoplastic parasagittal craniotomy	S-100 (++++)	Meningioma	Coronal suture	No
Reinshagen et al. 2016 [15]	39/F	Headache, neck tenderness, tinnitus, and dizziness/NA	Left frontal	4.3 × 3.3 × 1.4	NA	Iso to hypo	Heterogeneous hyper	Inhomogeneous	No	Left frontoparietal craniotomy	NA	Meningioma	Coronal suture	NA
Shrot et al. 2018 [16]	14/F	Confusion, altered mental status, rigidity, and fever/NA	Right frontal	2.4 × 2.0 × 1.4	Hyperdense/no calcification	Hypo	Hypo	Inhomogeneous	No	Right frontal craniotomy	NA	NA	None	NA
Raju et al. 2017 [17]	26/M	Headache and seizures/NA	Left frontotemporal	NA	Mixed density/calcification	NA	NA	Inhomogeneous	No	NA	NA	Meningioma	Coronal suture/squamosoparietal suture	NA
Colpan et al. 2003 [18]	40/F	Dizziness/3	Right frontoparietal	5.0 × 3.5 × 2.5	NA	Iso to hypo	Mixed	Inhomogeneous	No	Right frontoparietal craniotomy	NA	NA	Coronal suture/sutura sphenoehtmoidea	No
Wu et al. 1970 [19]	32/F	Speech difficulty and seizures/3	Left parietal	8.5 × 6.5 × 4.0	NA	NA	NA	NA	NA	Left parietal craniotomy	NA	NA	NA	NA
Hardy et al. 1978 [20]	22/F	Headache and intermittent dizziness/12	Left frontal	NA	NA	NA	NA	NA	NA	Left frontoparietal craniotomy	NA	NA	NA	No
Mapstone et al. 1983 [21]	17/M	Major motor seizure/NA	Right frontoparietal	8.0 × 5.0 × 5.0	Mixed density/calcification	NA	NA	NA	No	Right frontoparietal craniotomy	NA	NA	Coronal suture	NA
Nakazawa et al. 1993 [22]	16/F	Found coincidentally	Left parietal parasagittal	5.0 × 4.0	Mixed density/calcification	Hypo	Mixed hyper and iso	Inhomogeneous	Yes	Left parietal craniotomy	NA	NA	Coronal suture/sagittal suture	NA
Takano et al. 1997 [23]	53/F	Found coincidentally	Left frontotemporal	5.0 × 4.0 × 2.5	Hyperdense/NA	Heterogeneous hypo	Mixed	Inhomogeneous	No	NA	NA	NA	Suturae sphenofrontalis/sphenosquamosal suture	NA
Matz et al. 1981 [24]	20/M	Headache, vomiting, and blurred vision/8	Left frontoparietal	7.0 × 8.0	Hyperdense/calcification	NA	NA	NA	NA	Left frontotemporoparietal craniotomy	NA	NA	Coronal suture	No
Hong et al. 2005 [25]	18/M	Seizures/NA	Left frontal	3.0 × 3.0 × 4.0	Hyperdense/calcification	NA	NA	Inhomogeneous	No	NA	NA	NA	Coronal suture	NA
Lacerte et al. 1996 [26]	32/F	Headache and slight weakness and fine tremor of the left arm/NA	Right frontal	8.0 × 6.0 × 6.0	Mixed density/calcification	Hypo	Iso and hyper	NA	Yes	Right frontal craniotomy	NA	NA	Coronal suture	NA
Laghmari et al. 2007 [27]	50/M	Headache/NA	Right frontal	NA	Hyperdense/calcification	Iso	Hyper	Inhomogeneous	Yes	NA	S-100 (+)	NA	Coronal suture	No
Delgado-Lopez et al. 2007 [28]	18/M	Single generalized seizure/NA	Left frontal-parietal	8.0 × 6.0 × 2.0	NA	NA	NA	Inhomogeneous	Yes	Left frontal-parietal craniotomy	NA	NA	Coronal suture	No
Sullivan et al. 2019 [29]	31/M	Found coincidentally	Left frontal	2.4 × 1.9 × 0.9	NA	Hyper	Mixed hyper and hypo	Inhomogeneous	Yes	Left parietal craniotomy	NA	Meningioma	Sagittal suture	NA
Sugiura et al. 2015 [30]	45/F	Left-sided continuous headache /1	Left parietal	4.5	Iso to slightly high density mass/calcification	Hypo	Hyper	No	No	Frontoparietal craniotomy	Vimentin (+), S100P (+), EMA (-), Ki-67 (-)	NA	Squamosoparietal suture	No
Current case 1	37/F	Intermittent headache and dizziness/84	Right parietal	7.0 × 3.7 × 4.4	Hyperdense/calcification	Slightly hypo	Slightly hyper	Inhomogeneous	No	Right parietal-occipital craniotomy	CK (-), EMA (-), S-100 (+), vimentin (+), Brachyury (-), Ki-67 (0-1%)	Meningioma	Suture parieto-occipital	No
Current case 2	23/M	Found coincidentally	Right frontal	1.5 × 1.6 × 1.5	Hypodense/calcification	Uneven hypo	Uneven hyper	Inhomogeneous	Yes	Right frontal craniotomy	NA	Meningioma	Coronal suture	No
Current case 3	41/F	Dizziness/3	Left frontal	3.5 × 2.8 × 3.0	NA	Isointense	Mixed	Inhomogeneous	Yes	Left frontal craniotomy	P53 (-), Ki-67 (-)	Meningioma	Coronal suture	No
Current case 4	53/F	Found coincidentally	Left frontal	4.3 × 4.2 × 3.4	Hyperdense/calcification	Mixed	Mixed	Inhomogeneous	No	Left frontotemporal craniotomy	NA	Meningioma	Coronal suture	No
Current case 5	57/F	Right limb numbness/3	Left frontotemporal	6.3 × 4.0 × 5.2	Mixed density/calcification	Slightly hypo	Slightly hypo	Inhomogeneous	Yes	Left frontotemporal craniotomy	NA	Meningioma	Coronal suture	No

F: female; M: male; NA: not available; T1WI: T1-weighted imaging; T2-WI: T2-weighted imaging; Hyper: hyperintense; Hypo: hypointense; Iso: isointense.

## Data Availability

The data that support the findings of this study are available from the corresponding author upon reasonable request.

## Supplementary Materials

Supplementary Materials Figure S1: radiographic images and hematoxylin-eosin staining of Case 2. (a–d) Pre- and (e–g) postoperative radiographic images indicate a mass located in the right frontal region with cystic degeneration. (a) Coronal T1-weighted images (after gadolinium enhancement) demonstrate slight uneven enhancement. (b) Sagittal T1-weighted images demonstrate heterogeneous signals. (c) Axial T2 demonstrates uneven hyperintense and cystic degeneration in the lesion with no significant edema or mass effect in adjacent structures. (d) CT scan shows that the lesion was hypodense with calcification. (e–g) Postoperative CT scan shows that the tumor was completely removed (MRI was not available). (h) Hematoxylin-eosin staining of Case 2. Figure S2: radiographic images and hematoxylin-eosin staining of Case 3. (a–c) Pre- and (e–g) postoperative radiographic images indicate a mass located in the left frontal area with cystic degeneration. (a) Coronal T1-weighted images (after gadolinium enhancement) demonstrate marginal line-like enhancement. (b) Sagittal T1-weighted images demonstrate an isointense signal. (c) Axial T2 demonstrates mixed intense and cystic degeneration in the lesion with no significant edema or mass effect in adjacent structures. (d) Hematoxylin-eosin staining of Case 3. (e–g) Postoperative images show that the tumor was completely removed. Figure S3: radiographic images and hematoxylin-eosin staining of Case 4. (a–d) Pre- and (e–g) postoperative radiographic images indicate a mass located in the left frontal area with no cystic degeneration. (a) Coronal T1-weighted images (after gadolinium enhancement) demonstrate slight uneven enhancement. (b) Sagittal T1-weighted images demonstrate a mixed signal. (c) Axial T2 demonstrates mixed intensity with no significant edema or mass effect in adjacent structures. (d) CT scan shows that the lesion was hyperdense with calcification. (e–g) Postoperative images show that the tumor was completely removed. (h) Hematoxylin-eosin staining of Case 4. Figure S4: radiographic images and hematoxylin-eosin staining of Case 5. (a–d) Pre- and (e–g) postoperative radiographic images indicate a mass located in the left fronto-temporal lobe with cystic degeneration. (a) Coronal T1-weighted images (after gadolinium enhancement) demonstrate uneven puncture and linear enhancement. (b) Sagittal T1-weighted images demonstrate a slightly hypointense signal. (c) Axial T2 demonstrates slightly hypointense and cystic degeneration in the lesion with no significant edema or mass effect in adjacent structures. (d) CT scan shows that the lesion had mixed density with calcification. (e–g) Postoperative images show that the tumor was completely removed. (h) Hematoxylin-eosin staining of Case 5.
